# Mucosal Influenza Vector Vaccine Carrying TB10.4 and HspX Antigens Provides Protection against *Mycobacterium tuberculosis* in Mice and Guinea Pigs

**DOI:** 10.3390/vaccines9040394

**Published:** 2021-04-16

**Authors:** Mariia Sergeeva, Ekaterina Romanovskaya-Romanko, Natalia Zabolotnyh, Anastasia Pulkina, Kirill Vasilyev, Anna Polina Shurigina, Janna Buzitskaya, Yana Zabrodskaya, Artem Fadeev, Andrey Vasin, Tatiana I. Vinogradova, Marina A. Stukova

**Affiliations:** 1 Smorodintsev Research Institute of Influenza of the Ministry of Health of the Russian Federation, 197376 St. Petersburg, Russia; romromka@yandex.ru (E.R.-R.); pureska@mail.ru (A.P.); kirillv5@yandex.ru (K.V.); ann-polin@yandex.ru (A.P.S.); janna.buzitskaya@influenza.spb.ru (J.B.); zabryaka@yandex.ru (Y.Z.); afadeew@gmail.com (A.F.); vasin_av@spbstu.ru (A.V.); marina.stukova@influenza.spb.ru (M.A.S.); 2 Saint-Petersburg State Research Institute of Phthisiopulmonology of the Ministry of Health of the Russian Federation, 191036 St. Petersburg, Russia; zabol-natal@yandex.ru (N.Z.); vinogradova@spbniif.ru (T.I.V.); 3 Peter The Great St. Petersburg Polytechnic University, 195251 St. Petersburg, Russia

**Keywords:** *M. tuberculosis* multistage vaccine, TB10.4, HspX, influenza vector, mucosal immunization

## Abstract

New strategies providing protection against tuberculosis (TB) are still pending. The airborne nature of Mycobacterium tuberculosis (*M.tb)* infection assumes that the mucosal delivery of the TB vaccine could be a more promising strategy than the systemic route of immunization. We developed a mucosal TB vaccine candidate based on recombinant attenuated influenza vector (Flu/THSP) co-expressing truncated NS1 protein NS1(1–124) and a full-length TB10.4 and HspX proteins of *M.tb* within an NS1 protein open reading frame. The Flu/THSP vector was safe and stimulated a systemic TB-specific CD4+ and CD8+ T-cell immune response after intranasal immunization in mice. Double intranasal immunization with the Flu/THSP vector induced protection against two virulent *M.tb* strains equal to the effect of BCG subcutaneous injection in mice. In a guinea pig TB model, one intranasal immunization with Flu/THSP improved protection against *M.tb* when tested as a vaccine candidate for boosting BCG-primed immunity. Importantly, enhanced protection provided by a heterologous BCG-prime → Flu/THSP vector boost immunization scheme was associated with a significantly reduced lung and spleen bacterial burden (mean decrease of 0.77 lg CFU and 0.72 lg CFU, respectively) and improved lung pathology 8.5 weeks post-infection with virulent *M.tb* strain H37Rv.

## 1. Introduction

Tuberculosis (TB) is one of the most significant causes of morbidity and mortality among all infectious diseases. The World Health Organization (WHO) reported 10 million new cases of TB, a number that has been declining very slowly in recent years [1]. The evolution of multidrug-resistant (MDR) and extensively drug-resistant (XDR) tuberculosis and the high prevalence of TB-HIV co-infection have significantly complicated global TB control [2,3].

The only currently used TB vaccine, *Mycobacterium bovis* Bacillus Calmette–Guerin (BCG), is effective in the protection of newborns and infants against serious forms of disseminated childhood TB (miliary disease and meningitis) but has highly variable efficacy against pulmonary TB in adolescents and adults—the most widespread and transmissible form of TB [4,5]. The limited success of the BCG vaccine in the control of adult tuberculosis has been attributed at least in part to the fact that immune protection by BCG wanes over age and may last for only 10 to 15 years [6]. The reasons why BCG fails to protect against pulmonary TB could be due to co-infections (e.g., helminths) preventing the development of a protective immune response in the lungs or the lack of an adequate immune response in the respiratory tract after parenteral immunization [7].

Therefore, there is a critical need for new effective TB vaccines and immunization strategies for the different target populations [8]. To date, a number of novel TB vaccines are entering human clinical trials, including recombinant BCG, virus-vectored, and recombinant protein vaccine candidates [9]. The majority of currently developed TB vaccines are suitable for the parenteral route of delivery. Nevertheless, mucosal immunization is considered to provide better protection as it activates immunity in the respiratory tract— the route of entry of *M. tuberculosis* (reviewed in [10]). Currently, only two vector vaccine candidates, TB/FLU-04L (RIBSP, NCT02501421) and ChAdOx185A (University of Oxford, NCT04121494), utilize intranasal and aerosol vaccine administration to present TB antigens [1].

Considering the multistage process of TB pathogenesis, it is reasonable to assume that a vaccine based on a combination of M. tuberculosis immunodominant antigens, which are more stage-specific, may provide better protection against TB infection. Among the most studied TB antigens that can be used as targets for the activation of anti-mycobacterial protective immunity are secretory proteins related to the ESAT-6 family (Rv3875 (ESAT-6), Rv3874 (CFP-10) and Rv0288 (TB10.4)) and two proteins of the three-component Ag85 complex (Rv3804 (Ag85A) and Rv1886 (Ag85B)) [1]. Another promising antigen for vaccine development is a group of the latent associated proteins belonging to the DosR regulon [11]. For example, Rv2031c (HspX) was shown to be a promising TB vaccine target for the prevention and immunotherapy of tuberculosis [12,13]. Earlier studies demonstrated the potential of TB10.4 and HspX antigens as a multistage vaccine for TB prevention [14]. These antigens do not interfere with IGRA (interferon-gamma release assay) based diagnostic tools, employing ESAT-6, CFP-10, and TB7.7 *M.tb* proteins [15]. Moreover, TB10.4 and HspX are encoded by Bacillus Calmette-Guerin and, therefore, are useful for a prime-boost immunization scheme [16].

In the present study, we explored the characteristics of attenuated influenza vectors expressing TB10.4 and HspX antigens to induce mucosal immunity after intranasal administration. The vaccine candidate was evaluated for protective efficacy in mouse and guinea pig TB models using double immunization or BCG prime-boost immunization protocols.

## 2. Materials and Methods

### 2.1. Recombinant Influenza Viruses

**Figure 1:**
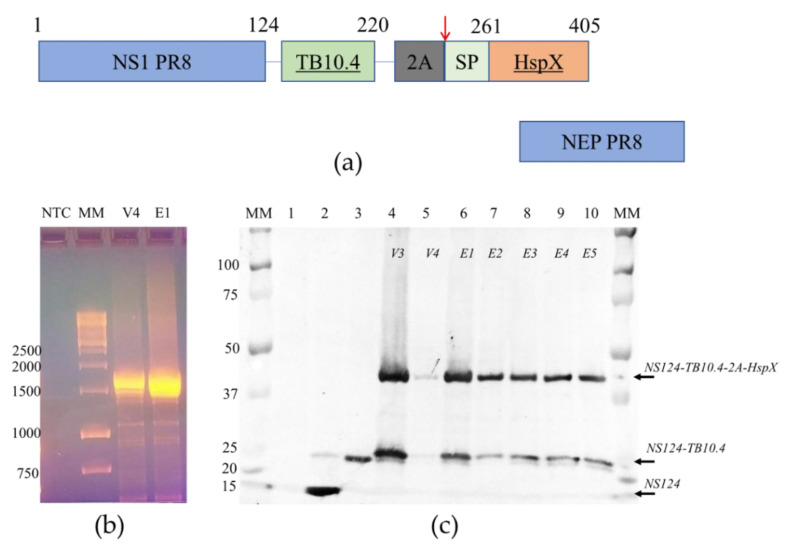
The structure and stability of the chimeric NS gene segment and proteins encoded. (**a**) The structure of NS gene OFRs of the Flu/THSP recombinant virus. The chimeric NS1 protein is synthesized from unspliced mRNA. TB10.4 antigen is directly linked to 124 amino acids of NS1; the HspX antigen is separated by the 2A “autoproteolysis” site and supplied with secretion signal peptide. NEP protein is synthesized from the spliced mRNA; (**b**) Full-length RT-PCR amplicons of the NS gene segment of the Flu/THSP virus passage variants V4 and E1. MM—marker in kb pairs; (**c**) Western blot detection of chimeric NS1 protein in cells infected by the Flu/THSP virus passage variants (lanes 4–10). MM—marker in kDa, lane 1—uninfected cells, lane 2—cells infected with empty virus vector A/PR8/NS124, lane 3—cells, infected with the A/PR8/NS124-TB10.4 recombinant virus carrying only TB10.4 antigen.

Recombinant viruses were generated by reverse genetics [17]. The ORF’s of *M. tuberculosis* TB10.4 and HspX antigens were inserted in the NS1 ORF of A/PR/8/34 virus after the 124 amino acid codon; the insert was terminated by a three-stop codon cassette, the NEP ORF was left unchanged (Figure 1). The designed chimeric NS gene, as well as the HA and NA genes of the A/PR/8/34 virus, were synthesized *de novo* (Evrogen, Russia) and cloned into the pHW2006 vector. The rest plasmids coding for the A/PR/8/34 virus backbone were kindly provided by Dr. Khairullin, Research Institute of Biological Safety Problems (Guardeyskiy uts, Kazakhstan). Vero cells (ATCC #CCL-81, USA) adapted to growth in serum-free medium (Gibco, Waltham, MA, USA) were transfected by Nucleofector system (Lonza #VCA-1003, EU) with eight plasmids (1 ug each) coding for 8 virus gene segments. After 72 h post-transfection, recombinant virus A/PR8-NS124-TB10.4-2A-HspX (Flu/THSP) was detected by the cytopathic effect and hemagglutination with 0.5% chicken erythrocytes. Next, several passages in limiting dilutions (cloning) were performed in Vero cells, followed by cloning in 10–12-day old chicken embryos (CE) (JSC “Poultry Farm Sinyavinskaya”, Priladozhsky village, Russia). Control of chimeric NS gene length (genetic stability) was done by RT-PCR with primers specific for NS UTR regions. The whole-genome sequencing of intermediate and final passage variants of recombinant Flu/THSP virus was done by NGS as described previously [18].

Recombinant influenza virus A/PR8/NS124-TB10.4, carrying only TB10.4 antigen in the NS1 ORF, was generated the same as described above except no multiple cloning was performed, and a stable clone was obtained after one passage in Vero cells and one in CE. Recombinant influenza virus A/PR8-NS124 (“empty vector”) was described earlier [19].

Virus infectious activity was determined by titration in CE, Vero, or MDCK cells (IRR, #FR-58) as described elsewhere. Infection was detected by positive hemagglutination, and the 50% infectious dose was calculated by the method of Reed-Muench [20]. The temperature-sensitive phenotype was defined by a decrease in infectious activity at 39 °C for more than 3.0 lg EID_50_ in comparison with 34 °C.

### 2.2. Antigen Expression Analyses

MDCK cells in 6-well plates were infected with recombinant viruses at moi = 10 TCID_50_/cell or mock-infected and incubated at 34 °C, 5% CO_2_. After 6 h, the cells were washed with DPBS (Biolot, Russia), detached by Trypsin-EDTA (Gibco, USA), and centrifuged. The cell spots were lysed by heating at 100 °C for 7 min in 80 μL of 1X Laemmli buffer (Bio Rad, Hercules, CA, USA). Proteins from 5 μL of samples were separated by SDS-PAGE and stained by Coomassie blue (Serva, EU) or transferred to nitrocellulose membrane (Bio Rad, USA) and probed with anti-NS1 1H7 monoclonal antibody [21].

To identify proteins by MALDI mass spectrometry analysis, the described PAGE was followed by tryptic hydrolyses in gel using the standard protocol. Briefly, a protein band of interest was cut from the gel, washed from the dye (twice by 30 mM NH_4_HCO_3_, 40% acetonitrile), and then dehydrated with 100% acetonitrile. After that, 2 uL of trypsin (20 ug/mL of Sequence grade modified trypsin porcine (Promega, Madison, WI, USA) in 50 mM NH_4_HCO_3_) was added to a dried piece of gel and incubated for 18 h, 37 °C. The reaction was stopped with 10% acetonitrile, 0.5% trifluoroacetic acid solution.

The resulting tryptic peptides were mixed with an HCCA matrix (alpha-cyano-4-hydroxycinnamic acid, Bruker, EU) and analyzed on a MALDI TOF/TOF mass spectrometer ultrafleXtreme (Bruker, EU) in positive ion detection mode. For each spectrum, 5000 laser pulses were summed. The amino acid sequence of recombinant protein was added to the local database. The identification of proteins was carried out using MASCOT [22] in order to access the SwissProt database [23] and a local database simultaneously. As variable modifications, the oxidation of methionine (M) was indicated. The maximum monoisotopic mass peak tolerance was limited to 50 ppm. Identification was considered reliable in the case of a protein score in excess of the threshold (*p* < 0.05).

### 2.3. Laboratory Animals

C57/black mice (male, 6–8-week-old) were obtained from the Biomedical Science Center (Stolbovaya, Russia). Guinea pigs (male, agouti, 200–250 g) were obtained from the Laboratory animal care facility Rappolovo (Rappolovo, Russia). All animal studies were done in accordance with the international recommendations (Directive 2010/63/EU) and the protocols approved by the Bioethics Committee at the Smorodintsev Research Institute of Influenza (Approval #61-2 dated 15 February 2018) and by the Bioethics Committee at the Saint-Petersburg State Research Institute of Phthisiopulmonology (Approval #4 dated 31 March 2018).

### 2.4. Safety and Immunogenicity Studies in Mice

Safety studies were performed in male C57/black mice. Animals were infected intranasally with 6.0 lg EID_50_ of recombinant virus in 30 μL total volume. Wild-type (wt) A/PR/8/34 (PR8) virus was used as pathogenic control (5.0 lg EID_50_/mouse), and intact mice served as the negative control group. Animal weight was monitored daily. On days 2, 3, and 5 post-infection, 6 animals per group (except intact) were euthanized, and the lungs were collected. 10% organ suspensions in DPBS were titrated in MDCK cells for the determination of the viral load.

To study vector immunogenicity, C57/black mice were immunized with 6.0 lg EID_50_ of virus per animal; intact mice served as controls. Analysis of the adaptive T-cell immune response was performed in spleens collected 8 days post-immunization. Single-cell suspensions were stimulated with 5 ug/mL of BCG for 24 h at 37 °C, 5% CO_2_. The GolgiPlug (BD Biosciences, USA) reagent was added 6 h before the end of stimulation. Medium alone and PMA/Ionomycin mix (both Sigma) were used as negative and positive controls, respectively. T-cell phenotyping was performed using the fluorochrome-conjugated antibody set containing CD4-PerCP-Cy5.5, CD8-PE/Cy7, CD62L-APC/Cy7, and CD44-BV421 (BioLegend, San Diego, CA, USA). Intracellular production of cytokines was assessed using fluorescently labeled antibodies IFNγ-FITC, IL2-PE, and TNFα-BV510 (BioLegend, USA). Staining for the detection of intracellular markers was performed using the Cytofix/Cytoperm kit (BD Biosciences, San Jose, CA, USA) according to the manufacturer’s instructions. Dead cells were identified using Zombie Red viability marker (BioLegend, USA). To block non-specific antibody binding, cells were pretreated with True Stain reagent (BioLegend, USA), containing antibodies to CD16/CD36. Data were collected on a Cytoflex flow cytometer (Beckman Coulter, Brea, CA, USA). The results were analyzed using the Kaluza Analysis 2.2 software (Beckman Coulter, USA).

### 2.5. Protection Study in Mice, Comparison to BCG

Protection study in mice was based on the previously established protocol [24]. Mice (C57/black, males) were immunized twice with 6.0 lg EID_50_ of recombinant influenza virus Flu/THSP intranasally (30 μL) at 3-week intervals. The comparative group of mice was immunized once with the *M. bovis* BCG strain by the subcutaneous injection of 5.0 lg CFU per animal. Control animals received a placebo—0.9% NaCl (Medpolimer, Russia). The challenge was performed 3 weeks post second immunization by the intravenous injection of 6.0 lg CFU of virulent *M. tuberculosis* strain Erdman or *M. tuberculosis* strain H37Rv, obtained from the collection of Scientific Centre for Expert Evaluation of Medicinal Products (Moscow, Russia). The severity of experimental tuberculosis was assessed 6 weeks after the challenge by evaluating bacterial load in lungs and spleen (n = 6), mass coefficients of lungs and spleen, lung damage score (n = 13) and histological evaluation of inflammation process in lungs (n = 6). Based on the number and size of TB specific lesions and presence the of areas of necrosis in the lungs, gross pathological scores were graded from 1 to 4 conventional units according to the following criteria: scanty small tubercles were estimated at 0.5 units (U), small tubercles (<5) as 1.0 U, numerous small tubercles (>5) as 1.5 U, occasional large tubercles as 1.75 U, small confluent tubercles and occasional large tubercles as 2.0 U, large tubercles (<10) as 2.25 U, numerous large tubercles (>10) as 2.5 U, numerous confluent tubercles as 2.75 U, tubercles with areas of necrosis as 3.0 U, numerous large necrotic tubercles as 4.0 U. In case of lung maceration by serous liquid, the index was increased by 0.25 to 1.0 U, depending on damage extent.

Phagocytosis was studied in the culture of peritoneal macrophages obtained from the lavage of the abdominal cavity of mice in relation to the cell suspension of the yeast Saccharomyces cerevisiae opsonized with mouse serum. Phagocytic activity was measured microscopically as the number of yeast cells digested by macrophages after 1.5 h of cultivation.

### 2.6. Protection Study in Guinea Pigs, Prime-Boost Immunization Scheme

Guinea pigs were primed with *M. bovis* BCG strain Pasteur by subcutaneous injection of 5.0 lg CFU per animal and boosted after 5.5 months with 6.0 lg EID_50_ of recombinant influenza virus Flu/THSP (30 μL intranasally). Control animals received placebo—0.9% NaCl (Medpolimer, Russia) instead of boost or both immunizations. The Challenge study was based on the previously established protocol [25]. Challenge was performed 6 weeks post the second immunization by subcutaneous injection of 9.0 lg CFU of *M. tuberculosis* strain H37Rv in the left inguinal area. The protection level was assessed 8.5 weeks after the challenge by evaluating the total damage score, mass coefficients of the lungs and spleen, bacterial load in lungs and spleen, as well as a histological evaluation of the inflammation process in the lungs. The total damage score was calculated as the summary index of exudate and progressive macroscopic lesions in lung tissue, liver, spleen, and lymph nodes (the maximum damage index for each organ tissue was equal to 4, and the maximum total score was equal to 20).

### 2.7. Mycobacterial Load

For the quantification of live mycobacteria load, tissue homogenates were titrated and cultured on solid Lowenstein-Jensen medium. Bacterial colonies were counted after 3 weeks of incubation at 37 °C. Titers were expressed as log10 of the mean colony forming units (lg CFU) per lung weight. The detection limit was equal to 2 × 10^3^ CFU. A decrease in the bacterial load of more than 0.5 lg CFU in comparison to the control group was considered a positive protective effect.

### 2.8. Histopathology

Lung tissues were fixed in 10% formalin (pH 7.0), followed by paraffin embedment. For histopathological studies, 5–6 mm sections were stained with hematoxylin and eosin. Images were captured using Olympus BX45 microscope (Olympus Corp., Tokyo, Japan) with camera and Olympus DP-Soft software package (Olympus Corp., Japan).

### 2.9. Statistical Analyses

Data analyses were performed using Prizm v.6.01 (GraphPad Software, San Diego, CA, USA) or the RStudio Desktop 1.0.153 (RStudio Inc., Boston, MA, USA). The experimental and control groups were compared using the suitable tests (Student’s *t*-test, Kruskal–Wallis test, and one-way or two-way ANOVA with the Bonferroni post-hoc-test), and the level of significance was assumed to be *p* < 0.05.

## 3. Results

### 3.1. Generation and Characterization of Stable Flu/THSP Recombinant Virus

**Figure 2:**
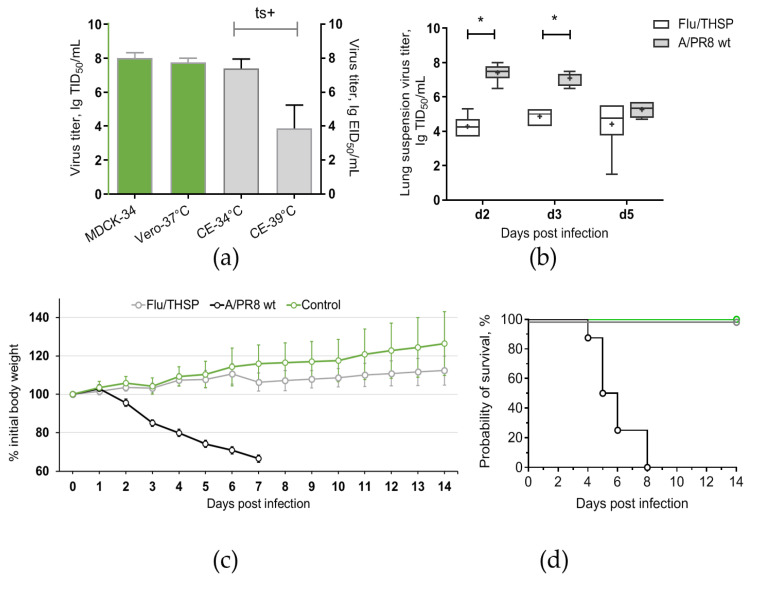
Reproductive properties of the Flu/THSP recombinant virus. (**a**) Virus replication in cell cultures and CE at permissive (34 °C and 37 °C) and elevated (39 °C) temperatures; (**b**) Virus replication in mouse lungs. Mice were infected with 6.0 lg EID_50_ of Flu/THSP virus (gray circles) or 5.0 lg EID_50_ of wild-type A/PR8 strain (black circles) and euthanized on days two, three, and five to determine viral load by titration of 10% lung suspension in MDCK cells. The data presented are mean values from six mice per group ± SD (error bars). Each dot represents an individual mouse. The difference between groups was analyzed by two-way ANOVA with the Bonferroni post-hoc-test (* *p* < 0.0001); (**c**,**d**) Weight dynamics and survival of mice infected with 6.0 lg EID_50_ of Flu/THSP virus (gray circles) or wild-type A/PR8 strain (black circles). Intact mice were used as control (green circles). Presented are group means with SD at indicated time post-infection (n = 8).

To generate attenuated recombinant influenza virus A/PR8/NS124-TB10.4-HspX (Flu/THSP) coding for *M. tuberculosis* TB10.4 and HspX antigens in NS1 ORF, we explored the previously reported scheme [24,26]. Therein, one of the antigens (TB10.4) is directly linked to shortened NS1, and the second (HspX) is inserted after the 2A “autoproteolysis” site and the IgK signal sequence (Figure 1a). The resulting length of the chimeric NS124-TB10.4-HspX gene segment counted 1720 bp versus 890 bp of wild-type NS. The virus obtained demonstrated low genetic stability, which might be caused by a long insert. In order to increase virus stability, we performed several cloning passages in Vero cells and CE to finally acquire the highly homogeneous virus population, which retained a full-length heterologous insert, as measured by RT-PCR of the NS segment (Figure 1b). Subsequent passaging in CE showed that the low multiplicity of infection (10 EID_50_/CE instead of conventional 100–1000 EID_50_/CE) could provide a high level of virus stability. The resultant Flu/THSP recombinant virus was obtained after five more passages in CE and checked by full-genome next-generation sequencing to be identical to the original virus. The recombinant virus Flu/THSP was shown to replicate to high titers (7.0–8.0 lg TCID_50_/mL) in Vero and MDCK cell cultures as well as in CE (Figure 2a). The 3.5 lg decrease in infectious activity in CE at elevated temperature (39 °C) indicated that the Flu/THSP recombinant virus has temperature-sensitive phenotype (Figure 2a).

### 3.2. Analysis of TB Antigen Expression Upon the Infection of Cells with Flu/THSP Recombinant Virus

Expression of heterologous antigens by a recombinant vector vaccine strain ensures their presentation to the immune system and the development of an appropriate immune response. We assessed the expression of TB antigens in MDCK cells infected with the Flu/THSP recombinant virus.

At first, we confirmed the synthesis of the full-length chimeric NS124-TB10.4-2A-HspX protein in infected cells and evaluated the degree of autoproteolysis by 2A site present in the construct. We performed Western blot analysis of infected cell lysates with anti-NS1 1H7 monoclonal antibody. For all passage variants of the Flu/THSP virus, the expression of the NS1 chimeric antigen with a double heterologous insert was detected, and the molecular weight of the corresponding band coincided with the theoretically calculated 45.1 kDa [27]. The expression of the NS1 chimeric antigen with a single heterologous insert TB10.4 appeared from “autoproteolysis” by the 2A site was also observed (calculated molecular weight 26.5 kDa). As controls, A/PR8/NS124 virus without the insert (calculated molecular weight 14.2 kDa) and A/PR8/NS124-TB10.4 virus with the insertion of the TB10.4 antigen alone (calculated molecular weight 24.7 kDa) were used (Figure 1c). No deletion or truncated variants of expressed proteins were observed.

**Figure A1:**
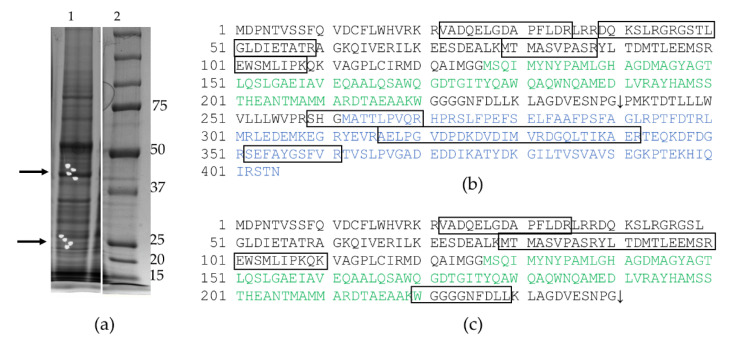
Mass spectrometric analyses of protein expression in cells infected with the Flu/THSP recombinant virus. (**a**) PAAG fragments intended to MS analysis (marked with arrows, see also (Figure 1c), lane 1—infected cells, lane 2—marker in kDa; (**b**) The amino acid sequence of NS1-TB10.4-HspX chimeric protein, which was reliably identified in PAAG zones corresponding to the molecular weight of 45 kDa (score/threshold was equal to 108/61); the protein fragments identified in the spectrum are outlined. (**c**) The amino acid sequence of NS1-TB10.4 chimeric protein, which was reliably identified in PAAG zones corresponding to the molecular weight of 26 kDa (score/threshold was equal to 70/61); the protein fragments identified in the spectrum are outlined. Sequences corresponding to TB10.4 antigen and HspX antigen are marked green and blue, respectively. The 2A-site is marked with an arrow.

Secondly, we tried to confirm the expression of TB antigens by Western blot with anti-TB10.4 (ABIN361292) or anti-HspX (sc-58169) antibodies but failed to detect any specific bands (not shown). Therefore, we performed a mass spectrometric analysis of proteins in the gel slices corresponding to the bands detected by the anti-NS1 antibody. It should be noted that major proteins (for example, actin) were excluded during the preliminary processing of the spectra. In the zone of electrophoretic mobility approximately corresponding to 45 kDa, fragments of the full-length NS1-TB10.4-HspX protein were reliably identified. In the 26 kDa zone, fragments of NS1-TB10.4 protein were reliably identified (Figure A1). Therefore, we confirmed the intracellular expression of TB antigens TB10.4 and HspX upon the infection of cells by Flu/THSP recombinant virus.

### 3.3. Safety and Immunogenicity of Flu/THSP Recombinant Virus in Mice

Safety of Flu/THSP recombinant vector was evaluated by intranasal inoculation of C57 mice at a dose 6.0 lg EID_50_ per animal. The comparison was made with a non-attenuated A/PR8 strain with full-length NS1 protein given at a dose 5.0 lg EID_50_/mouse. As expected, mice inoculated with wild-type virus exhibited significant weight loss and death. In contrast, mice from Flu/THSP virus group did not suffer from weight loss and showed significantly reduced viral load in lungs on days two, three, and five after inoculation (Figure 2b,c). Thereby, the Flu/THSP influenza vector demonstrated an attenuated phenotype in mice.

**Figure 3:**
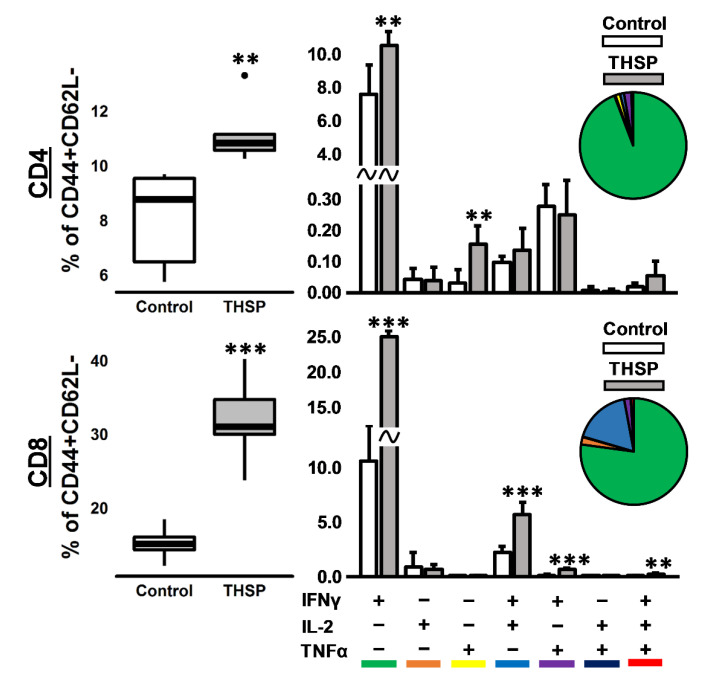
Immune response in mice after intranasal immunization with Flu/THSP recombinant virus. Antigen-specific effector memory T-cellular immune response in the spleen was assessed eight days after immunization by estimating the number of CD4/8+ CD44+CD62L- effector memory (EM) T-cells expressing IFNγ, IL-2, and TNFα after 24 h stimulation with BCG. Box plots represent the total amount of cytokine-producing EM T-cells. Bar-plots represent the average percentage of each cytokine-producing population within the total EM T-cells subset ± SD. Pie charts show the average percent of each cytokine-producing population within a total subset of cytokine-producing cells. The differences between groups were analyzed using the Student’s *t*-test (** *p* < 0.01, and *** *p* < 0.001, n = 5).

To assess the immunogenicity of the Flu/THSP influenza vector, we measured the cytokine response of immunized and control (intact) animals to BCG, which contains both antigens TB10.4 and HspX. The stimulation of splenocytes from mice previously immunized with Flu/THSP recombinant virus-induced a strong cytokine response, mediated by CD8+ and CD4+ effector memory (EM) T-cells (Figure 3). IFNγ+ single-positive CD8+ EM T-lymphocytes made up the most abundant population of antigen-specific T-cells, but the populations of polyfunctional cytokine-producers (IFN-γ+IL-2+, IFN-γ+TNF-α+, and IFN-γ+IL-2+TNF-α+) were also presented. Therefore, the single intranasal immunization with A Flu/THSP efficiently induces systemic TB antigen-specific memory T-cell response.

### 3.4. Protection Studies of Flu/THSP in Mouse Model in Comparison to BCG

The protective efficacy of mucosal immunization with recombinant vector Flu/THSP was first explored in comparison to single subcutaneous BCG vaccination in a mouse TB model. Animals were vaccinated and then challenged with virulent *M. tuberculosis* strains Erdman or H37Rv. After six weeks of infection, the animals were sacrificed, and their organs were analyzed for tuberculosis damage and mycobacteria load.

**Figure 4:**
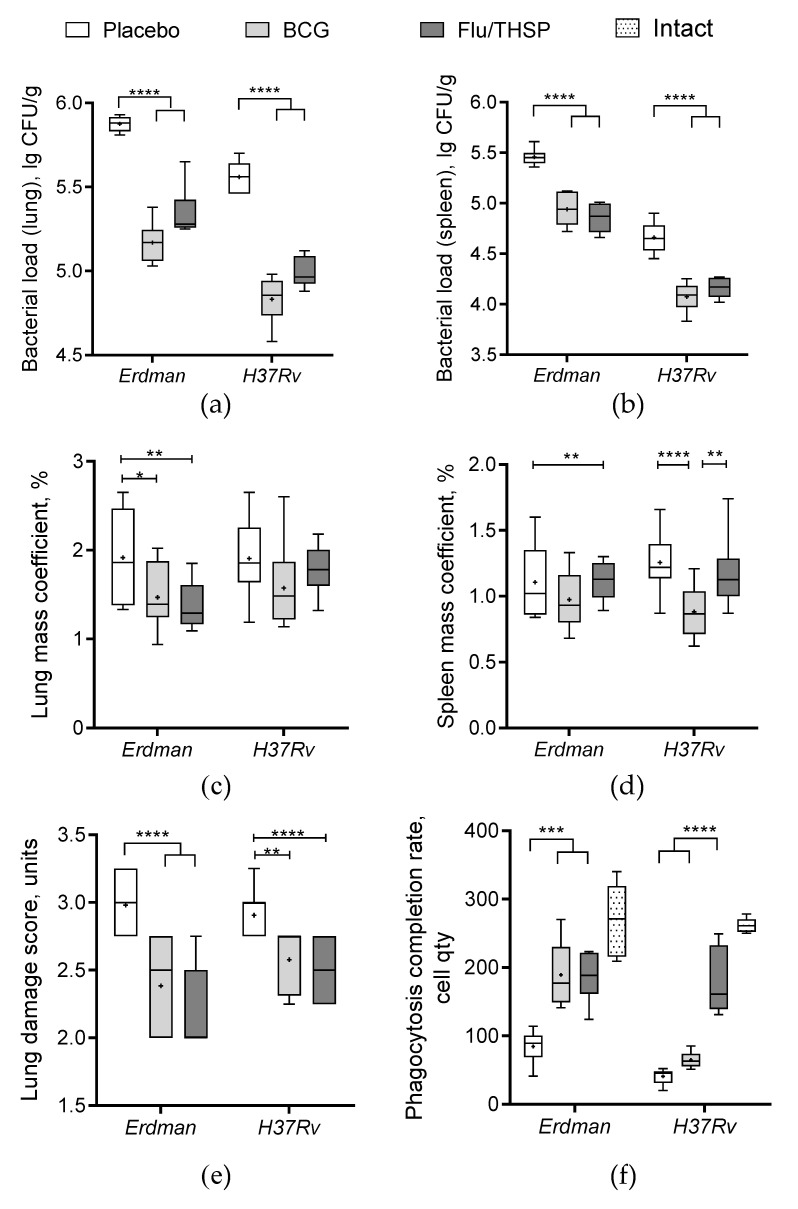
Protective efficacy of Flu/THSP in a mouse model of TB infection. Protective efficacy of Flu/THSP vaccinated mice six weeks post TB infection. Mice were immunized with Flu/THSP or BCG or placebo (indicators are shown on top), challenged with virulent *M. tb* Erdman or H37Rv strain as indicated, and analyzed six weeks post-infection. (**a**,**b**) Bacterial load in lungs and spleen was measured by titration of tissue homogenates; (**c**,**d**) Lungs and spleen mass coefficients were estimated as the percentage of organ weight relative to body weight; (**e**) The lung damage score was quantified in conventional units as described in Section 2.6; (**f**) Phagocytosis completion rate of model yeast cells was measured. Statistical analyses were performed by two-way ANOVA, and the results of the Bonferroni post-hoc-test for a pairwise comparison are presented by asterisks (* *p* < 0.05, ** *p* < 0.01, *** *p* < 0.001, and **** *p* < 0.0001).

The robust protective effect was demonstrated accessing the level of bacterial load in lung and spleen, which was ≥0.5 lg CFU lower in the vector or BCG immunized animals than in the placebo group, wherein the difference was statistically significant (Figure 4a,b). In addition, the increase in relative lung mass coefficient was observed for the placebo group in comparison to immunized groups; however, the difference was less prominent (Figure 4c). Protection was also notable by visual lung tissue analysis revealing a decrease in the specific inflammation process for BCG and vector immunized mice compared to placebo (Figure 4e). The level of protection was similar between the BCG and Flu/THSP groups.

We also checked the phagocytosis activity of peripheral macrophages, which was inhibited in infected unvaccinated mice compared to the intact group (Figure 4f). We observed that both BCG and Flu/THSP immunization significantly improved the phagocytosis completion rate. Moreover, the phagocytosis rate for mice immunized with Flu/THSP vector during infection with virulent *M.tb* H37Rv strain was almost comparable to that of intact animals.

**Figure 5:**
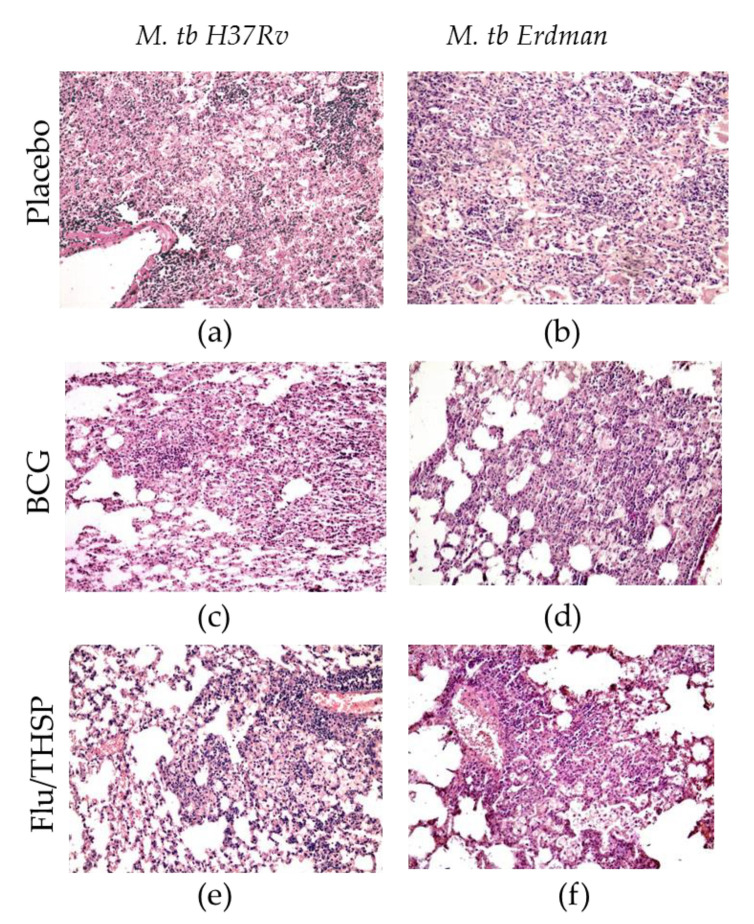
Protective efficacy of Flu/THSP against lung pathology in a mouse model of TB infection. Representative hematoxylin and eosin (H&E) stained lung histological sections of vaccinated and control mice (indicated on the left) after infection with highly virulent *M. tb* H37Rv or *M. tb* Erdman strains (indicated above), magnification is 300X. (**a**,**b**)—merged foci of specific infiltration without a clear spatial orientation of the cells; (**c**,**d**)—small foci of specific infiltration, lymphocytes, foamy macrophages, epithelioid cells, and single neutrophilic leukocytes; (**e**,**f**)—small alveolar-macrophage infiltrates, perivascular lymphohistiocytic infiltration.

The study of the lung histological sections of mice infected with *M. tuberculosis* H37Rv revealed a widespread specific lung lesion, represented by confluent infiltration foci with blurred boundaries without a clear spatial orientation of cells. In all cases (6/6 animals), specific infiltrates reduced the airiness of the lung tissue by more than 30% of the section area (representative photo shown in Figure 5a). After BCG vaccination, the prevalence of specific lesions in the lung tissue was significantly lower than in the infection control group (Figure 5c). A decrease in the airiness of the lung tissue by more than 30% of the sectional area was noted in 1/6 cases. Specific infiltration lost its confluent character, and in 5/6 mice was represented by separate small foci of infiltration. After prophylactic immunization with Flu/THSP, the histological picture of tuberculous inflammation corresponded to that in animals vaccinated with BCG, in all cases, it was represented by small alveolar-macrophage infiltrates without signs of exudation, in which alveoli and intercellular septa were infiltrated by lymphocytes, macrophages, and epithelioid cells (Figure 5e). Nearly the same difference in histological damage was observed in vaccinated and placebo mice infected with the virulent *M. tuberculosis* Erdman strain (Figure 5b,d,f).

### 3.5. Protection Studies of Prime-Boost Immunization Protocol in the Guinea Pig Model

Protective efficacy of prime-boost vaccination protocol with first BCG immunization and second Flu/THSP immunization was accessed in comparison to single BCG vaccination or placebo in the guinea pig model. Animals were vaccinated and then challenged with the virulent *M. tuberculosis* strain H37Rv. After 8.5 weeks of the infection process, guinea pigs were sacrificed, and their organs were analyzed for tuberculosis damage and bacterial load.

**Figure 6:**
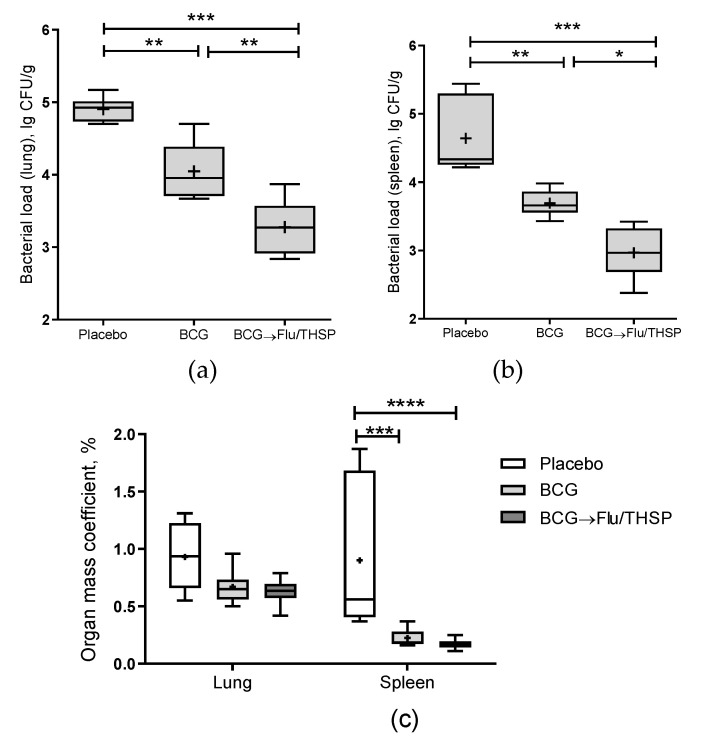
Protective efficacy of BCG-prime → Flu/THSP boosts immunization in a guinea pig model of TB infection. Vaccinated guinea pigs were challenged with virulent *M.tb* H37Rv strain and analyzed 8.5 weeks post TB infection. (**a**) Bacterial load in the lungs and (**b**) spleen was measured by the titration of tissue homogenates; (**c**) Organ mass coefficients were estimated as the percentage of organ weight relative to body weight. Statistical significance was measured by two-way ANOVA (familywise *p* < 0.0001) with the Bonferroni post-hoc-test for a pairwise comparison (* *p* < 0.05, ** *p* < 0.01, *** *p* < 0.001, and **** *p* < 0.0001).

The robust protective effect was demonstrated accessing the level of bacterial load in lung and spleen, which was significantly lower in prime-boost vaccinated animals than in BCG group (mean decrease by 0.77 lg CFU and 0.72 lg CFU in the lungs and spleen, respectively) (Figure 6a,b). Furthermore, the relative mass coefficient of the spleen was significantly lower for prime-boost vaccinated guinea pigs than for BCG or placebo vaccinated animals (Figure 6c).

**Figure 7:**
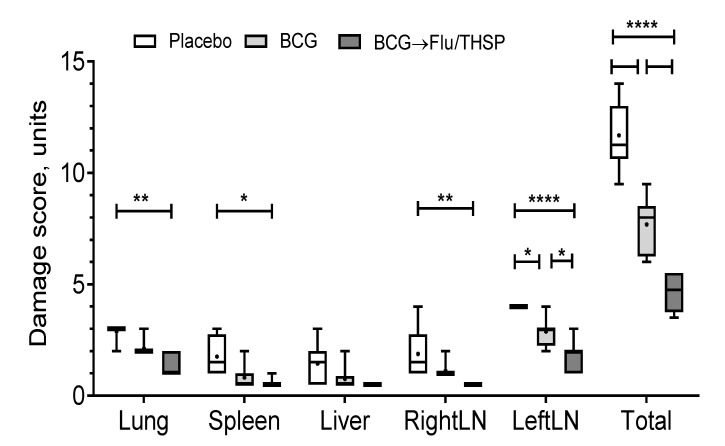
Protective efficacy of BCG-prime → Flu/THSP boosts immunization against tuberculosis damage in a guinea pig model. Damage score of tuberculosis-specific inflammation in vaccinated and control guinea pigs 8.5 weeks post-challenge with the *Mtb* H37Rv strain. The score of specified organ damage was estimated visually as described in Materials and Methods. Statistical significance was measured by two-way ANOVA (familywise *p* < 0.0001) with the Bonferroni post-hoc-test for pairwise comparison (* *p* < 0.05, ** *p* < 0.01, **** *p* < 0.0001).

Visual analysis of specific inflammation process in tissues taken 8.5 weeks post-challenge showed the least damage of parenchymal organs, and inguinal lymphatic nodes by tuberculosis infection in guinea pigs vaccinated by prime-boost protocol, which was significantly lower than in the placebo vaccinated group and in BCG vaccinated group (Figure 7). The detected difference in the total damage score was also due to the lower level of lung damage in the prime-boost vaccinated guinea pigs who demonstrated the near full absence of specific lesions and secondly due to the decreased damage of regional inguinal lymphatic nodes, which were completely free of necrosis.

**Figure 8:**
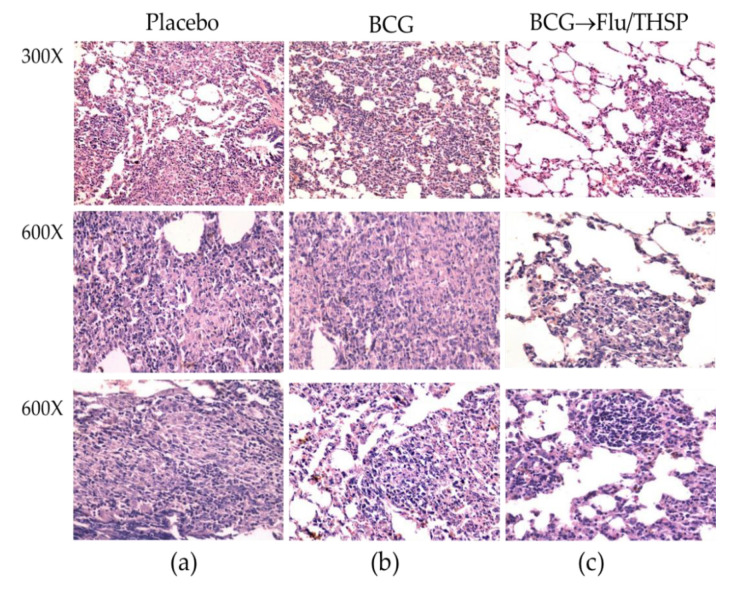
Protective efficacy of BCG-prime → Flu/THSP boosts immunization against lung pathology in a guinea pig model of TB infection. Representative H&E stained lung histological sections of vaccinated and control guinea pigs (indicated above) after infection with *M.tb* H37Rv strain, magnification is indicated at the left. (**a**)—intensive inflammation foci with a large number of degrading neutrophils and nuclear debris are demonstrated; merging granulomas are characterized by large areas of caseous necrosis; (**b**)—the particular focus of specific infiltration can be found; (**c**)—the small peribronchiolar focus of specific infiltration can be found.

Microscope examination of the lung histological sections indicated that animals in the prime-boost vaccinated group exhibited a delay of the specific damage process presented by a decrease in inflammation foci size and the number and lesser spectrum of visible lesions (Figure 8c). No large granulomas were found. The foci of specific inflammation were small, did not merge, had no signs of caseous or liquefactive necrosis, and were predominately located perivascularly or peribronchially causing no significant reduction in the lung tissue airiness. Alveoli and interalveolar septa infiltrations were presented by lymphocytes and macrophages and did not contain epithelioid cells or neutrophils.

In contrast, the analysis of lung histological sections of the BCG vaccinated group revealed more developed granulomas with predominantly epithelioid composition. Moreover, in two of six cases, neutrophil clusters accumulations, areas of nuclear detritus, and foci of caseous necrosis were found (Figure 8b).

## 4. Discussion

An ideal vaccine against tuberculosis has not yet been developed. The first problem lies in the difficulty of choosing a protective antigen for inclusion in a vaccine. To overcome the innate immune system at the different stages of infection, *M. tuberculosis* expresses various proteins that can be used as vaccine targets [28]. Most anti-tuberculosis vaccines that are currently ongoing clinical trials target the early expressed antigens. However, latency-associated antigens, such as HspX, have recently emerged in the development of anti-tuberculosis vaccines [29].

New approaches of multistage tuberculosis vaccines combine early antigenic proteins with latency-associated antigens. Vaccines comprising the latency-associated proteins can be either given prophylactically to naive persons or given as a post-exposure vaccine to prevent reactivation. Andersen and colleagues have developed a multistage subunit vaccine H56 comprising both early Ag85B and ESAT-6 and latency-associated Rv2660c antigens [30]. This vaccine is currently in an ongoing phase 2b clinical trial (NCT03512249).

Although BCG, a live attenuated vaccine strain, could express and secrete thousands of proteins, it could not confer a significant immune response to the latency-associated antigens belonging to the *M. tuberculosis* DosR regulon [31]. Thus, screening of protective antigens expressed at different stages of *M. tuberculosis* infection is a particularly important task that can be useful for the development of new vaccine candidates.

In the present study, we obtained an influenza vector expressing *M. tuberculosis* full-size TB10.4 and HspX protein sequences replacing the C-terminal half of the NS1 protein. The Flu/THSP vector displayed genetic stability and was shown to possess the high yield phenotype in most production substrates (CE, Vero, MDCK). The intracellular expression of inserted TB proteins was confirmed by mass spectrometry. Western blot with specific antibodies failed to detect any bands, possibly due to the conformational discrepancies between TB antigens used to generate antibodies and the TB antigens linked to the NS1 within the chimeric protein. However, the confirmation biases were not critical as we suppose linear-epitope-based T-cell response as the main protection correlate, yet this is a question for further studies.

The attenuated phenotype of the Flu/THSP recombinant influenza vector was confirmed in vivo. Mice that received intranasally 6.0 lg EID_50_ of the recombinant virus did not exhibit weight loss and had a significantly decreased viral load in the lungs compared to the *wt* A/PR/8/34 (H1N1) virus. Thus, the vector was nonpathogenic in mice due to impaired function of the NS1 protein to antagonize interferon response [32,33]. Attenuation in the lungs was even more pronounced than in the case of the empty A/PR8-NS124 vector lacking foreign sequences [19,32], suspecting the possible influence of the long heterologous insert in NS1 on the vector attenuation. In addition, we found the Flu/THSP vector acquired a *ts* phenotype by losing the capacity to replicate at 39 °C, which was also observed for other NS1 mutant influenza viruses [33]. Temperature sensitivity can be considered as an independent attenuation marker and mechanism, restricting virus growth in the lower respiratory tract (as for live attenuated influenza vaccine strains [34]). Importantly, the C-terminal deletion of NS1 is not only responsible for the attenuated phenotype but also results in stimulating an efficient adaptive T-cell-mediated immune response due to enhanced induction of pro-inflammatory cytokines at the site of application [19,35].

Previously, Florido et al. assessed properties of influenza vector carrying TB10.4 epitope (3–11) in NA ORF as a vaccine candidate; however, it did not induce enough protection, probably because of insufficient activation of antigen-specific CD4+ T-cells [36]. Earlier influenza viruses with shortened NS1 protein were shown to exhibit self-adjuvant properties [37] and to be more effective in the presentation of viral antigens due to the intensification of the dendritic cell migration [19], which can explain the advantages of our approach. We demonstrated the appearance of CD4+ and CD8+ effector memory T-cells preferably expressing IFNγ+ single-positive CD8+ T-lymphocytes and minor populations of polyfunctional cytokine-producers in the spleen of the intranasally vaccinated mice.

The efficacy of vaccination with the Flu/THSP vector against tuberculosis infection in mice was tested in our experiments using intravenous challenge with virulent *Mtb* Erdman and *Mtb* H37Rv strains. We found that double influenza Flu/THSP vector immunization could provide a substantial level of protection comparable to the BCG vaccine after two intranasal applications.

Protective efficacy of the prime-boost immunization scheme (BCG → Flu/THSP) was studied in the guinea pig TB model. Guinea pigs are generally accepted as the most suitable model for tuberculosis vaccines. The protection level induced by BCG was significantly improved by one intranasal boost immunization with the Flu/THSP vector. Compared to the BCG-only vaccinated guinea pigs, prime-boost immunized animals had significantly lower bacterial counts in their lungs and spleens and showed improved lung histology pictures.

## 5. Conclusions

Our study presented the scope and perspective of the mucosal immunization with attenuated influenza vector producing early expressed (TB10.4) and latency-associated (HspX) antigens to provide protection against pulmonary tuberculosis. The study supported the conception of a multistage vaccine against tuberculosis. The protection was achieved in two animal models and immunization schedules assuming independent application or combination with BCG in the prime-boost strategy. The underlying immunological mechanism of protection is a subject for further study.

## Data Availability

The data presented in this study are available on reasonable request from the corresponding author.
